# p16^INK4a^-induced senescence is disabled by melanoma-associated mutations

**DOI:** 10.1111/j.1474-9726.2008.00422.x

**Published:** 2008-10

**Authors:** Sebastian Haferkamp, Therese M Becker, Lyndee L Scurr, Richard F Kefford, Helen Rizos

**Affiliations:** Westmead Institute for Cancer Research, University of Sydney at Westmead Millennium Institute, Westmead HospitalWestmead, NSW 2145, Australia

**Keywords:** CDK4, CDK6, melanoma, naevi, p16^INK4a^, senescence

## Abstract

The p16^INK4a^-Rb tumour suppressor pathway is required for the initiation and maintenance of cellular senescence, a state of permanent growth arrest that acts as a natural barrier against cancer progression. Senescence can be overcome if the pathway is not fully engaged, and this may occur when p16^INK4a^ is inactivated. p16^INK4a^ is frequently altered in human cancer and germline mutations affecting p16^INK4a^ have been linked to melanoma susceptibility. To characterize the functions of melanoma-associated p16^INK4a^ mutations, in terms of promoting proliferative arrest and initiating senescence, we utilized an inducible expression system in a melanoma cell model. We show that wild-type p16^INK4a^ promotes rapid cell cycle arrest that leads to a senescence programme characterized by the appearance of chromatin foci, activation of acidic β-galactosidase activity, p53 independence and Rb dependence. Accumulation of wild-type p16^INK4a^ also promoted cell enlargement and extensive vacuolization independent of Rb status. In contrast, the highly penetrant p16^INK4a^ variants, R24P and A36P failed to arrest cell proliferation and did not initiate senescence. We also show that overexpression of CDK4, or its homologue CDK6, but not the downstream kinase, CDK2, inhibited the ability of wild-type p16^INK4a^ to promote cell cycle arrest and senescence. Our data provide the first evidence that p16^INK4a^ can initiate a CDK4/6-dependent autonomous senescence programme that is disabled by inherited melanoma-associated mutations.

## Introduction

The *INK4a/ARF* locus, situated on chromosome band 9p21, is one of the most frequently altered sequences in human cancer and germline mutations affecting this locus have been linked to melanoma incidence in approximately 39% of melanoma-prone families (Goldstein *etal*., 2006b). The lifetime risk of melanoma in p16^INK4a^ germline mutation carriers ranges from 58% in Europe to 91% in Australia by the age of 80 (Bishop *etal*., 2002). This locus encodes two potent, but distinct tumour suppressor proteins; the cyclin-dependent kinase inhibitor, p16^INK4a^ (Serrano *etal*., 1993) and the p53 activator p14ARF (Quelle *etal*., 1995). Both proteins are critically important in the regulation of cell cycle progression and senescence (reviewed in Sharpless, 2005; Collado *etal*., 2007). p14ARF blocks proliferation by inhibiting the p53 ubiquitin ligase hdm2, to stabilize and activate p53 (Pomerantz *etal*., 1998; Stott *etal*., 1998; Zhang *etal*., 1998) and ARF-null mouse embryonic fibroblasts do not senesce (Kamijo *etal*., 1997). p16^INK4a^ promotes cell cycle arrest by inhibiting the kinase activities of the cyclin D-dependent kinases, CDK4 and CDK6, to maintain the retinoblastoma protein, Rb in its hypophosphorylated, antiproliferative state (Serrano *etal*., 1993). The progressive accumulation of p16^INK4a^ is associated with the onset of replicative senescence in primary human epithelial cells (Alcorta *etal*., 1996; Brenner *etal*., 1998) and ectopic p16^INK4a^ expression induces growth arrest that phenotypically resembles cellular senescence in human diploid fibroblasts (Zhu *etal*., 1998; McConnell *etal*., 1999) and in *INK4a/ARF*-deficient murine melanocytes (Sviderskaya *etal*., 2002). Furthermore, p16^INK4a^-deficient human diploid fibroblasts and melanocytes, isolated from melanoma-prone individuals with inactivating mutations affecting both *INK4a* alleles, undergo delayed senescence (Sviderskaya *etal*., 2003; Brookes *etal*., 2004; Jones *etal*., 2007) and are readily immortalized by the introduction of the telomerase reverse transcriptase (Sviderskaya *etal*., 2003).

Cellular senescence can be triggered by multiple mechanisms including induction of the *INK4a/ARF* locus, telomere attrition, DNA damage, oxidative damage and the aberrant proliferative signals of oncogenes (reviewed in Collado & Serrano, 2006). Once established, senescence permanently limits cellular proliferation and protects against the development of malignant cancer. Accordingly, senescent cells are abundant in premalignant lesions of the skin, the lung and the pancreas whereas they are almost completely absent in malignant tumours (Collado *etal*., 2005). Senescent cells have been identified, both *in vitro* and *in vivo*, using a series of markers (reviewed in Collado & Serrano, 2006; Campisi & d’Adda di Fagagna, 2007). Increased activity of acidic β-galactosidase, termed senescence-associated β-galactosidase (SA-β-gal) is the most widely accepted marker of senescence cells (Dimri *etal*., 1995). More recently, the appearance of DAPI-stained heterochromatic regions, known as senescence-associated heterochromatic foci, which result in the stable repression of some E2F target genes are involved in the irreversible growth arrest associated with senescence (Narita *etal*., 2003). These foci are enriched for histone H3 modified at lysine 9 as well as its binding partner heterochromatin protein-1γ (HP-1γ) (Narita *etal*., 2003). Several other markers of senescence have also been described and validated, including the CDK inhibitor p15^INK4b^, an anti-apoptotic bcl-2 member, Mcl-1 and the transcription factor, Dec1. Morphological changes such as cell enlargement, vacuolization and cell flattening are also typical of senescent cells (Collado *etal*., 2005).

Although the role of p16^INK4a^ in initiating senescence is well documented (Vogt *etal*., 1998; McConnell *etal*., 1999; Dai & Enders, 2000; Sviderskaya *etal*., 2002), there is a paucity of data on the mechanisms underlying p16^INK4a^-induced senescence. Moreover, little is known regarding the ability of melanoma-associated p16^INK4a^ germline mutations to initiate and maintain cellular senescence. This is particularly important as individuals carrying p16^INK4a^ mutations have increased susceptibility to melanoma, and usually display larger, more numerous and dysplastic naevi (Gruis *etal*., 1995; Bennett & Medrano, 2002). There is persuasive evidence that melanocytic naevi are growth-arrested, senescent lesions, and it is likely that p16^INK4a^, which is widely expressed in naevi, contributes to establishing cellular senescence (Michaloglou *etal*., 2005; Gray-Schopfer *etal*., 2006). This permanent growth arrest would be an efficient barrier to melanoma development that may not be triggered by melanoma-associated p16^INK4a^ variants.

To examine the autonomy and mechanism of p16^INK4a^-induced senescence, we utilized a melanoma cell model with inducible, physiological levels of p16^INK4a^ expression and compared the impact of wild-type p16^INK4a^ expression to that of two functionally distinct p16^INK4a^ melanoma-associated mutants. The R24P mutation alters a highly conserved residue in the first ankyrin repeat, is closely linked with familial melanoma in at least 11 melanoma-prone kindreds (Della Torre *etal*., 2001; Mantelli *etal*., 2002; Goldstein *etal*., 2006a) but behaved as wild-type p16^INK4a^ in CDK6-binding assays (Harland *etal*., 1997; Jones *etal*., 2007). This mutant was shown to be defective in binding CDK4 (Harland *etal*., 1997) and inducing senescence in human fibroblasts (Jones *etal*., 2007), but has not previously been analysed in cells of melanocytic lineage. The A36P variant identified in an Australian family (Holland *etal*., 1999) is impaired in promoting cell cycle arrest (Becker *etal*., 2001), but there is currently no data on its interaction with CDK4 and CDK6 *in vivo*. We show that wild-type p16^INK4a^ induced a rapid proliferative arrest that was associated with cell enlargement, vacuolization and appearance of heterochromatic foci. This senescence programme was not triggered by stress to the endoplasmic reticulum or DNA damage and was not activated by the mis-sense melanoma-associated mutants, R24P and A36P. Transient expression of CDK4 or CDK6, but not the downstream kinase CDK2 overcame p16^INK4a^-induced senescence programme, and although Rb was critical to p16^INK4a^-mediated arrest, it was not required for p16^INK4a^-induced cell enlargement and vacuolization. This work confirms p16^INK4a^-driven senescence is intimately linked to CDK4/6-inhibition and cell cycle arrest, and indicates that the melanoma-associated mis-sense p16^INK4a^ mutants are unable to initiate an effective CDK4/6-dependent senescence programme.

## Results

### Impact of induced wild-type p16^INK4a^ expression

**Fig. 1:**
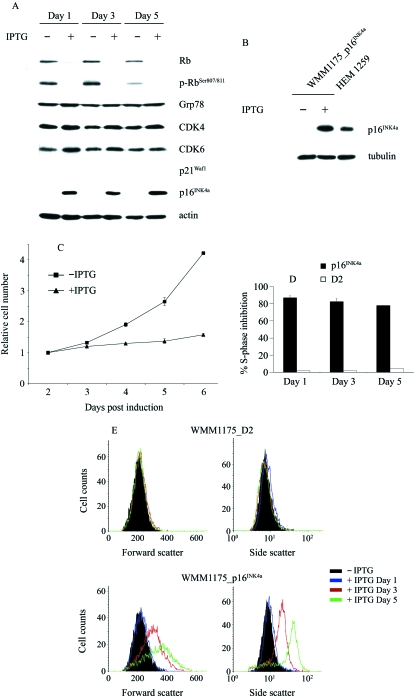
Induced expression of p16^INK4a^ inhibits Rb phosphorylation, limits cell proliferation and alters cell morphology. (A) Expression of the indicated proteins was determined by Western blot at 1, 3 and 5 days after treatment of WMM1175_p16^INK4a^ cells with 4 mm IPTG. (B) Accumulation of p16^INK4a^ in IPTG-treated (5 days) WMM1175_p16^INK4a^ cells compared with levels of endogenous p16^INK4a^ in normal, actively proliferating human neonatal epidermal melanocytes (HEM 1259). (C) The impact of induced p16^INK4a^ expression on the proliferation of the WMM1175_p16^INK4a^ cells was determined over a 5-day induction period using the MTS assay. The results shown are expressed as the average ± standard deviation of at least two independent experiments performed in triplicate. (D) The percentage of cells in S-phase after induction of p16^INK4a^ for up to 5 days was determined by flow cytometry. S-phase inhibition was calculated from at least two independent induction experiments. Percentage S-phase inhibition in the IPTG-treated parental WMM1175_D2 cells, which expresses the lac repressor, but not the *p16^INK4a^* transgene, is also shown. (E) The impact of IPTG-exposure on the size (Forward scatter) and granularity (Side scatter) of the WMM1175_p16^INK4a^ melanoma cells and the parental WMM1175_D2 cell line was investigated using flow cytometry on unfixed cells. These results are representative of at least two independent experiments.

To evaluate the influence of wild-type p16^INK4a^ accumulation on cell proliferation and senescence, the WMM1175 melanoma cell line, which is *INK4a/ARF-* and *p53*-null (Rizos *etal*., 1999) was engineered to express wild-type p16^INK4a^. In this WMM1175_p16^INK4a^ cell line, p16^INK4a^ expression was induced with 4 mm isopropyl β-D-1-thiogalactopyranoside (IPTG). Accumulation of p16^INK4a^ was detected in the WMM1175_p16^INK4a^ cells 24 h post-induction, and this was maintained for the 5 days of continuous IPTG exposure (Fig. 1A). The level of p16^INK4a^ accumulation in the WMM1175_p16^INK4a^ clone was comparable to p16^INK4a^ expression in normal, actively proliferating human epidermal melanocytes at passage 10 (Fig. 1B). The accumulation of p16^INK4a^ led to the decreased levels of phosphorylated Rb (p-Rb^Ser807/811^), the loss of total Rb protein expression (Fang *etal*., 1998), and a slight increase in the accumulation of CDK4 and CDK6. The p53-target p21^Waf1^ was not detectable and the endoplasmic reticulum-stress response, which is required for H-RAS-induced senescence (Denoyelle *etal*., 2006) was not activated by p16^INK4a^, as determined by the lack of induction of the endoplasmic reticulum-stress sensor Grp78 (BiP) (reviewed in Gething, 1999) (Fig. 1A). Furthermore, the DNA damage checkpoint, which mediates oncogene-induced senescence (Bartkova *etal*., 2006; Di Micco *etal*., 2006), was not induced by p16^INK4a^ induction as there was no evidence of increased DNA damage foci in p16^INK4a^-expressing cells, as marked by H2AX phosphorylation (data not shown).

As previously demonstrated (Becker *etal*., 2001), accumulation of p16^INK4a^ potently inhibited the proliferation of the WMM1175 cell line (Fig. 1C), and this was associated with a rapid arrest in the G1-phase of the cell cycle with a concomitant S-phase inhibition that was maintained over the 5-day induction period (Fig. 1D). To ensure that IPTG alone did not affect cell proliferation, the parental WMM1175_D2 cell line, which expresses the lac repressor but not p16^INK4a^, was treated as the WMM1175_p16^INK4a^ clones and no changes were observed in proliferation (data not shown) or cell cycle distribution (Fig. 1D).

**Fig. 2:**
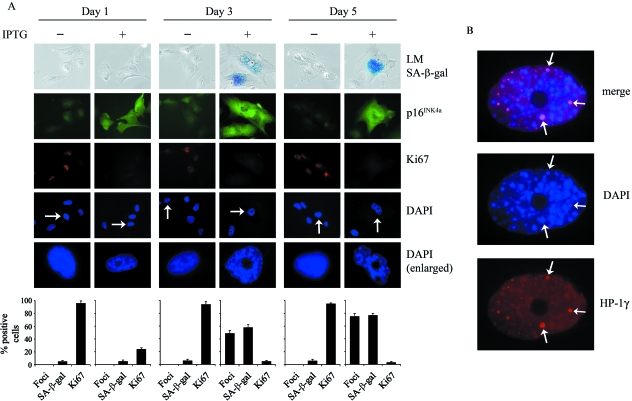
Impact of induced p16^INK4a^ expression on the cellular senescence programme. (A) WMM1175_p16^INK4a^ cells were exposed to 4 mm IPTG over a 5-day period. The accumulation of p16^INK4a^, cell proliferation (Ki67), chromatin condensation (DAPI) and the appearance of SA-β-gal was analysed. Cells enlarged to show DAPI-stained chromatin foci are indicated with arrows. Cell counts for each of these markers are shown as histograms, which correspond to the average ± standard deviation of at least two independent induction experiments from a total of at least 500 cells. LM, light microscopy. (B) Representative examples of p16^INK4a^-induced chromatin condensation (DAPI) and costaining for HP-1γ as surrogates for senescence-associated heterochromatin foci (indicated by arrows).

To further examine the p16^INK4a^-induced arrest, we analysed two key markers of senescence: cell size and vacuolization. Whereas IPTG treatment did not alter the phenotype of the parental WMM1175_D2 cells (Fig. 1E), it caused an obvious increase in the size and granularity, a marker of vacuolization, in the WMM1175_p16^INK4a^ cells (Fig. 1E). These p16^INK4a^-induced morphological changes were confirmed using microscopy. As shown in Fig. 2A, at 3 days post-induction the cells induced for p16^INK4a^ expression had adopted characteristics of senescent cells, appearing enlarged and flattened. These cells were negative for the proliferation marker Ki67 and acquired SA-β-gal activity (77 ± 3% of p16^INK4a^-induced cells stained positive for SA-β-gal at 5 days post-induction). These senescence features occurred as late markers of p16^INK4a^ function, and appeared 2 days later than p16^INK4a^-induced cell cycle arrest, which was evident within 24 h post p16^INK4a^ induction (see Fig. 1D). The uninduced WMM1175_p16^INK4a^ cells (Fig. 2A) and IPTG-treated parental WMM1175_D2 cells (data not shown) did not display a senescence phenotype and the majority of these cells stained positive for Ki67.

To further investigate the response of the WMM1175 melanoma cells to p16^INK4a^ we analysed senescence-associated heterochromatic foci by immunostaining with DAPI and HP-1γ. We observed a dramatic increase in the appearance of nuclear foci at 3 and 5 days post-IPTG treatment; 49 ± 5% and 75 ± 5% of p16^INK4a^-induced cells stained positive for nuclear foci at 3 and 5 days post-induction, respectively, with the appearance of large, prominent, often irregular shaped nuclei (Fig. 2A). The accumulation the HP-1γ, within these nuclear foci confirmed that they are senescence-associated heterochromatic foci (Fig. 2B). Taken together our results confirm that wild-type p16^INK4a^ can induce senescence in WMM1175 melanoma cells in a p53- and p21^Waf1^-independent manner.

### Impact of melanoma-associated p16^INK4a^ mutations on melanoma cell senescence

**Fig. 3:**
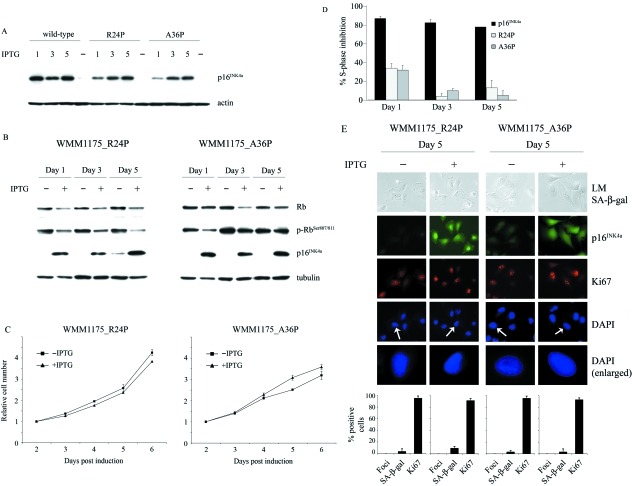
Melanoma-associated p16^INK4a^ mutants fail to induce cell cycle arrest. (A) Expression of wild-type p16^INK4a^, R24P and A36P mutant proteins in WMM1175 melanoma cell clones was induced with 4 mm IPTG over a 5-day induction period and compared using immunoblotting. (B) Expression of p16^INK4a^, total Rb, Ser^807/811^-phosphorylated Rb and actin was determined 1, 3 and 5 days after treatment of WMM1175_R24P and WMM1175_A36P cells with 4 mm IPTG. (C) The impact of induced mutant p16^INK4a^ on the proliferation of the WMM1175_R24P and WMM1175_A36P cells was determined over the 5-day induction period using the MTS assay. The results shown are expressed as the average ± standard deviation of at least two independent experiments performed in triplicate. (D) The percentage of cells in S-phase after induction of p16^INK4a^ or a melanoma-associated p16^INK4a^ mutant in the WMM1175 cells was determined by flow cytometry. The percentage S-phase inhibition was calculated from at least two independent induction experiments. (E) Expression of the melanoma-associated variants R24P or A36P was induced in the WMM1175 melanoma cells and the impact on cell morphology (LM), proliferation (Ki67), chromatin condensation (DAPI) and SA-β-gal activity was analysed over a 5-day period. Representative examples of the 5-day IPTG induction point are shown. Cells enlarged to show DAPI-stained chromatin foci are indicated with arrows. Cell counts for the cell cycle markers are shown as histograms, which correspond to the average ± standard deviation of at least two independent induction experiments from a total of at least 500 cells. LM, light microscopy.

Although it has been shown that wild-type p16^INK4a^ can promote an autonomous senescence programme (Dai & Enders, 2000), there has been no detailed analysis on the impact of melanoma-associated mutations on this programme. This is particularly relevant as there are significant variations in the penetrance of p16^INK4a^ mutations for melanoma (Berwick *etal*., 2006) and this may relate to loss of specific functions, including the induction of senescence. We analysed two well-defined and common p16^INK4a^ mutants that segregate with disease in high-risk melanoma families. These mutant proteins were also selected because they displayed expression levels comparable to the wild-type p16^INK4a^ protein in the inducible WMM1175 melanoma cell model (Fig. 3A).

All clones expressing mutant p16^INK4a^ proteins were analysed as the wild-type WMM1175_p16^INK4a^ clone in cell proliferation and senescence assays. The R24P mutant, which retains CDK6 inhibitory activity, partially inhibited Rb phosphorylation and slightly diminished the levels of total Rb over the 5-day induction period (Fig. 3B). In contrast, the A36P mutant had no consistent impact on Rb levels or its phosphorylation status (Fig. 3B). Expression of the p16^INK4a^ mutations (R24P and A36P) had no long-term inhibitory effect on WMM1175 cell proliferation (Fig. 3C) and this correlated closely with the consistently weaker inhibition of S-phase induced by the mutants when compared with the sustained S-phase inhibition and G1 arrest observed when the wild-type p16^INK4a^ protein was induced (Fig. 3D). Furthermore, these mutant p16^INK4a^ proteins produced no detectable changes in cell size and morphology (data not shown), had no impact on heterochromatic foci and did not induce SA-β-gal activity (Fig. 3E).

### p16^INK4a^-induced arrest and senescence requires inhibition of CDK4 and CDK6

**Fig. 4:**
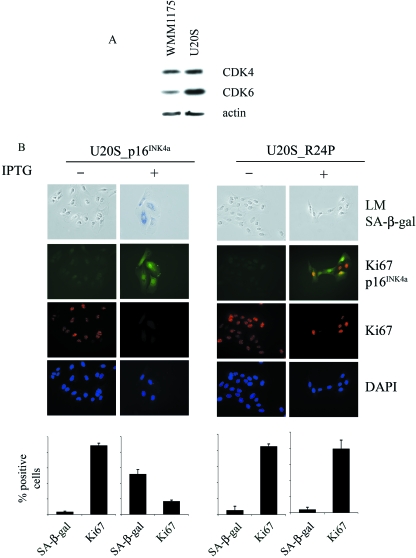
The melanoma-associated p16^INK4a^ R24P mutant fails to induce cell cycle arrest or senescence in U20S cells. (A) Expression of CDK4, CDK6 and actin was compared in the U20S and WMM1175 cells. Band intensities were determined by densitometric measurements using a phosphoimager (Molecular Dynamics). (B) Stable pools of U20S cells expressing inducible forms of wild-type p16^INK4a^ or R24P were exposed to 4 mm IPTG over a 5-day period. Representative examples of the 5-day IPTG induction time point are shown. The accumulation of p16^INK4a^, cell proliferation (Ki67) and the appearance of SA-β-gal was analysed. Cell counts for these markers are shown as histograms, which correspond to the average ± standard deviation of at least two independent induction experiments from a total of at least 500 cells. LM, light microscopy.

Given that the R24P variant, which binds and inhibits CDK6, but not CDK4 (Jones *etal*., 2007), was still incapable of promoting arrest, we hypothesized that inhibition of both CDK4 and CDK6 was required for p16^INK4a^-induced arrest and senescence. After screening a panel of human cancer cell lines for CDK4 and CDK6 expression (data not shown), the U20S cell line was selected, as it accumulated approximately threefold higher levels of CDK6 compared to the WMM1175 cell line (Fig. 4A). U20S cells were engineered to inducibly express the wild-type p16^INK4a^ or the R24P mutant protein. As expected, expression of wild-type p16^INK4a^, but not R24P, arrested U20S cells and promoted their senescence. p16^INK4a^-induced senescence in the U20S cells was associated with cell enlargement and SA-β-gal activity, but not DNA heterochromatic foci (Fig. 4B).

**Fig. 5:**
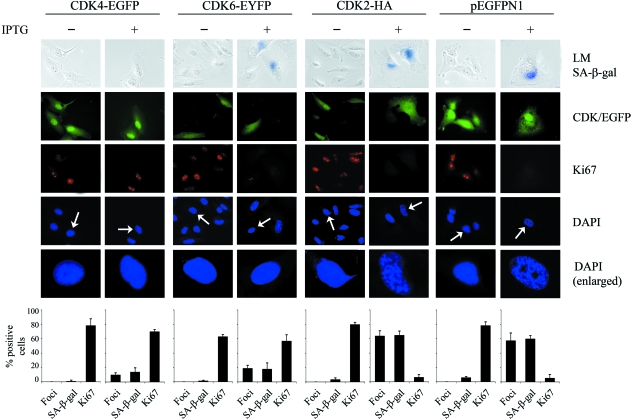
CDK4 and CDK6 inhibition is critical to p16^INK4a^-induced senescence. WMM1175_p16^INK4a^ cells were transfected with *CDK4-EGFP*, *CDK6-EYFP*, *CDK2-HA* or vector DNA (only the *pEGFPN1* vector control is shown here), as indicated. Approximately 6 h post-transfection, cells were treated with PBS (–) or induced for p16^INK4a^ expression with 4 mm IPTG (+). At 72 h post-induction, cells were stained for transgene expression (CDK/GFP), markers of senescence (SA-β-gal, DAPI) and proliferation (Ki67), as indicated. Cell counts for each of these markers are shown as histograms, which correspond to the average ± standard deviation of at least two independent induction experiments from a total of at least 300 cells.

Furthermore, when either CDK4 or CDK6 was ectopically expressed in the WMM1175_p16^INK4a^ cell line, induced expression of p16^INK4a^ failed to inhibit cell proliferation and did not induce senescence. In particular, in the presence of ectopic CDK4 or CDK6 expression, p16^INK4a^ did not promote cell enlargement, heterochromatic foci or SA-β-gal activity (Fig. 5). In contrast, WMM1175_p16^INK4a^ cells transiently transfected with vector only and induced for wild-type p16^INK4a^ expression showed all the characteristic markers of senescence (Fig. 5).

To ensure that p16^INK4a^-mediated cell cycle arrest was specifically overcome by expression of its CDK4 and CDK6 binding partners, we also transiently introduced CDK2, a kinase that accelerates and augments CDK4/6-initiated Rb hyperphosphorylation (reviewed in Johnson & Walker, 1999; Sherr, 1993). Ectopically expressed CDK2 did not overcome the ability of p16^INK4a^ to induce cell cycle arrest or senescence (Fig. 5). These data confirm that the inhibition of both CDK4 and CDK6 kinase activity is required for p16^INK4a^-mediated cell cycle arrest and senescence. More importantly, they suggest that all known functions of p16^INK4a^, including the induction of chromatin condensation and p16^INK4a^-mediated changes in cell morphology and size, depend on CDK4/6 binding and inhibition.

### The Rb protein is the critical downstream target of p16^INK4a^

**Fig. 6:**
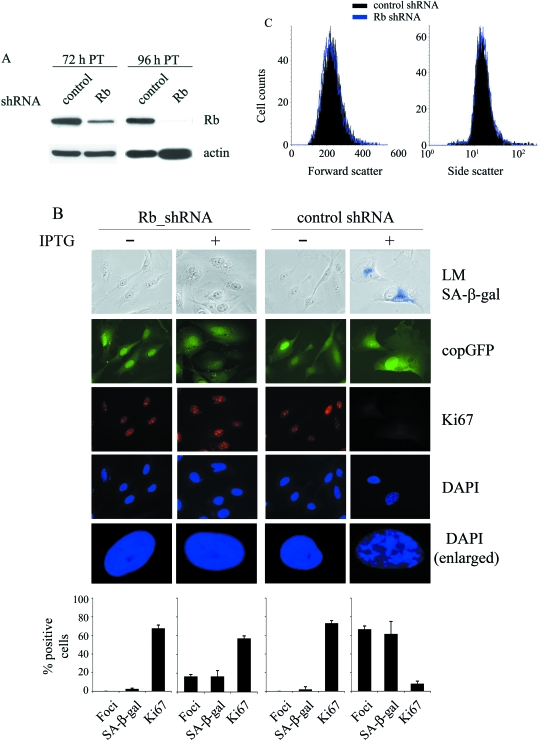
Silencing Rb expression does not promote an arrest or senescence response. (A) WMM1175 melanoma cells were transduced with a control shRNA or an Rb-specific silencing molecule, as indicated. The efficiency of transduction was controlled with co-expression of copGFP and was consistently above 90%. At 72 h and 96 h post-transduction (PT), cells were harvested and protein expression analysed using SDS-PAGE with the indicated antibodies. (B) WMM1175_p16^INK4a^ cells were transduced with a control or a Rb-specific shRNA molecule and approximately 96 h post-transduction the cells were treated for 3 days with IPTG (+) or PBS (–) and stained for markers of transduction (copGFP), senescence (SA-β-gal, DAPI) and proliferation (Ki67), as indicated. Cell counts for each of these markers are shown as histograms, which correspond to the average ± standard deviation of at least two independent induction experiments from a total of at least 300 cells. (C) The impact of Rb silencing on the size (Forward scatter) and granularity (Side scatter) of the WMM1175 melanoma cells was investigated, 96 h post-transduction, using flow cytometry on paraformaldehyde fixed cells. These results are representative of at least two independent experiments.

It is well established that the downstream impact of p16^INK4a^-mediated inhibition of CDK4 and CDK6 activity is the hypophosphorylation and activation of Rb, as shown in Fig. 1A. It has also been recognized that p16^INK4a^ accumulation promotes the rapid disappearance of Rb (Serrano *etal*., 1997; Fang *etal*., 1998; Ausserlechner *etal*., 2005), and we observed Rb loss in both the WMM1175 (Fig. 1A) and U20S cells (data not shown). Considering that Rb loss coincided with cell cycle arrest and occurred earlier than the onset of senescence (Rb loss and arrest were detected 24 h post p16^INK4a^ induction, whereas senescence was detected 72 h after p16^INK4a^ expression was induced; see Figs 1A and 2A), it was important to establish whether Rb depletion alone (with no p16^INK4a^ expression) promoted cell cycle arrest and senescence. Silencing of Rb with an Rb-specific silencing molecule, 72 h or 96 h post-transduction (Fig. 6A), did not in itself promote cell cycle arrest or senescence as judged by the continued proliferation of Rb shRNA-transduced WMM1175 (data not shown) and WMM1175_p16^INK4a^ cells (Fig. 6B). These Rb-null cells did not stain positive for SA-β-gal activity, did not form DNA foci (Fig. 6B) and did not enlarge (Fig. 6C). Thus, down-regulation of Rb expression alone does not inhibit cell cycle proliferation nor does it promote senescence. More importantly, induced p16^INK4a^ could only promote cell cycle arrest and cellular senescence, as judged by acquired Ki67 staining and SA-β-gal activity and DNA foci formation, in the presence of Rb (Fig. 6B).

**Fig. 7:**
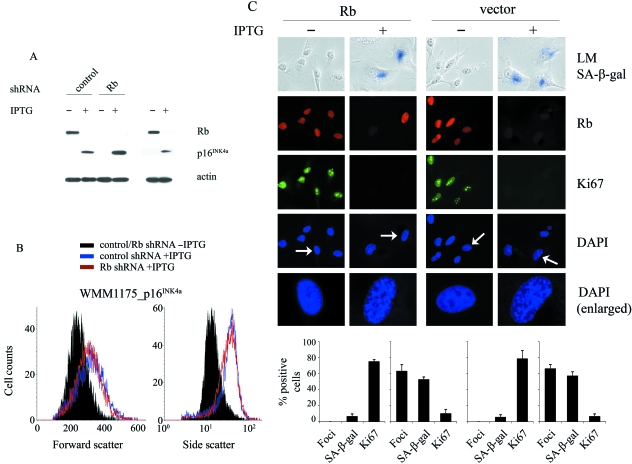
p16^INK4a^-induced arrest and senescence requires the expression of Rb.WMM1175_p16^INK4a^ melanoma cells were transduced with a control shRNA or an Rb-specific silencing molecule, as indicated. The efficiency of trasduction was controlled with the co-expression of copGFP and was consistently above 90%. At 96 h post-transduction cells were treated with PBS (–) or 4 mm IPTG (+) and 72 h post-induction cells were analysed. (A) Cells were harvested and protein expression analysed using SDS-PAGE with the indicated antibodies. (B) The impact of 72 h of p16^INK4a^ induction on the size (Forward scatter) and granularity (Side scatter) of the WMM1175_p16^INK4a^ melanoma cells was investigated, 96 h post-transduction with a control or Rb-specific shRNA molecule, using flow cytometry on paraformaldehyde fixed cells. These results are representative of at least two independent experiments. (C) WMM1175_p16^INK4a^ cells were transiently transfected with an *Rb* or empty expression plasmid, and approximately 6 h post-transfection the cells were treated for three days with IPTG (+) or PBS (–) and stained for Rb, markers of senescence (SA-β-gal, DAPI) and proliferation (Ki67), as indicated. Cell counts for each of these markers are shown as histograms, which correspond to the average ± standard deviation of at least two independent induction experiments from a total of at least 300 cells.

Although loss of Rb did not initiate senescence and Rb was required for p16^INK4a^-mediated senescence it was important to clarify whether Rb was the critical downstream target for all p16^INK4a^ functions. This was particularly relevant as it has been suggested that CDK4 and CDK6 may phosphorylate as yet unidentified substrates (Ruas *etal*., 2007), and we have now shown that these binding partners are critical for p16^INK4a^-induced senescence. Thus, the WMM1175_p16^INK4a^ cells were transduced with a control shRNA or an Rb-specific silencing molecule and 96 h post-transduction the cells were induced for wild-type p16^INK4a^ expression. Analysis of these cells, 72 h after p16^INK4a^ induction, revealed that Rb expression remained effectively silenced and the cells remained inducible for p16^INK4a^ expression (Fig. 7A). Intriguingly, the ability of p16^INK4a^ to increase cell size and granularity did not require Rb, and p16^INK4a^ induced these distinctive changes in cell morphology regardless of Rb status (Fig. 7B).

Considering that depletion of Rb occurs after the onset of p16^INK4a^-induced arrest but prior to the onset of p16^INK4a^-induced senescence (see Fig. 1), it was possible that reinstating the expression of Rb could influence p16^INK4a^-induced senescence. As shown in Fig. 7(C), however, when Rb expression was transiently reintroduced into the WMM1175_p16^INK4a^ cells, p16^INK4a^ retained its activity and effectively promoted cell cycle arrest followed by senescence. Thus, p16^INK4a^-induced cell cycle arrest and senescence requires the complete inhibition of CDK4 and CDK6 activity and the transient activation of Rb. The subsequent loss of Rb expression appears incidental to p16^INK4a^-mediated arrest and senescence, although we are investigating the precise mechanism of Rb loss. Furthermore, the p16^INK4a^-driven inhibition of CDK4 and CDK6 promotes changes in cell morphology independent of Rb, suggesting additional kinase targets may contribute to the activity of p16^INK4a^.

## Discussion

p16^INK4a^ is a highly penetrant melanoma tumour suppressor that regulates cell cycle progression by inhibiting the kinase activities of cyclin D-associated CDK4 and CDK6 (Serrano *etal*., 1993). Active binary cyclin D-CDK complexes initiate Rb phosphorylation driving cells towards DNA replication in S-phase. Although the CDK inhibitory functions of p16^INK4a^ are well described, the mechanisms underlying p16^INK4a^-mediated senescence are poorly understood and the relative contribution of p16^INK4a^-induced senescence to its role as a tumour suppressor has not been addressed.

A few studies have examined the impact of p16^INK4a^ on the senescence of human dermal fibroblasts and melanocytes derived from rare melanoma-prone individuals carrying germline mutations in both *INK4a/ARF* alleles. These p16^INK4a^-deficient cells were resistant to oncogenic RAS-induced senescence (Huot *etal*., 2002; Jones *etal*., 2007) and had an extended, but finite lifespan that terminated with senescence (Sviderskaya *etal*., 2003; Brookes *etal*., 2004). Interpretation of these results is complicated, however, by the possible contribution of defective p14ARF (Huot *etal*., 2002; Sviderskaya *etal*., 2003; Brookes *etal*., 2004), which is known to confer a growth advantage when silenced (Voorhoeve & Agami, 2003). Moreover, there is considerable variability in the different cell strains with regard to their lifespan (Brookes *etal*., 2004; Jones *etal*., 2007), p16^INK4a^ expression (Beausejour *etal*., 2003), chromosomal stability (Sviderskaya *etal*., 2003) and inducibility of p16^INK4a^ by the RAS oncogene (Jones *etal*., 2007). To avoid some of these confounding effects, silencing molecules have been applied to deliberately and specifically ablate p16^INK4a^. Nevertheless, the data remain inconclusive; in most, but not all reports, p16^INK4a^ deficiency modestly extended the replicative lifespan of cells but did not impair senescence (Bond *etal*., 1999; Voorhoeve & Agami, 2003; Denoyelle *etal*., 2006). p16^INK4a^ was also not essential for H-RAS-induced melanocyte senescence (Denoyelle *etal*., 2006), although it was required for RAS-induced fibroblast senescence (Bond *etal*., 1999; Huot *etal*., 2002).

As an alternative strategy, we applied an inducible melanoma cell model to thoroughly characterize the p16^INK4a^ senescence pathway, with a particular emphasis on the analysis of well-established markers of senescence. We then examined the impact of two melanoma-associated mis-sense p16^INK4a^ mutations on the senescence of this melanoma cell model. By utilizing an inducible cell clone we eliminated cell-related variations and manipulated the induction of p16^INK4a^ and melanoma-associated p16^INK4a^ variants, to obtain near-physiological expression levels. As expected, the wild-type p16^INK4a^ protein promoted rapid cell cycle arrest that was associated at later time points with the onset of senescence and the appearance of classic senescence markers, including enlarged cells with heterochromatic foci and SA-β-gal activity. As expected, these markers proved useful in combination, as none are specific or persistent in all senescence cells (Collado & Serrano, 2006). In fact, U20S cells induced to express wild-type p16^INK4a^ acquired SA-β-gal activity, showed a large increase in cellular size but did not feature condensed chromatin. Although senescence-associated heterochromatic foci are associated with the silencing of E2F-1 genes (Narita *etal*., 2003), it is evident that they are late markers of senescence, occur later than E2F-1 target gene silencing (data not shown) and can be absent in highly vacuolized and arrested cells (Denoyelle *etal*., 2006).

The mis-sense p16^INK4a^ variants, R24P and A36P, failed to inhibit proliferation and to initiate senescence. The R24P mutation was able to slightly reduce the hyperphopshorylation of Rb, presumably because it retains CDK6 inhibitory activity but this was not sufficient to maintain a G1 arrest. This is consistent with data indicating that this is a highly penetrant melanoma-susceptibility mutant that has been identified in at least eight melanoma-prone families worldwide (Goldstein *etal*., 2006b). Furthermore, it reinforces that CDK4, rather than CDK6 (Shennan *etal*., 2000), is the critical kinase in melanoma. CDK4 germline mutations have been identified in eight melanoma-prone families worldwide (Zuo *etal*., 1996; Soufir *etal*., 1998; Molven *etal*., 2005; Pjanova *etal*., 2007; Soufir *etal*., 2007) and these disrupt the interaction between p16^INK4a^ and CDK4 (Zuo *etal*., 1996). Mouse embryonic fibroblasts derived from CDK4^R24C/R24C^ mice (CDK4^R24C^ is resistant to p16^INK4a^ inhibition) (Rane *etal*., 2002) and human diploid fibroblasts overexpressing CDK4 have an extended lifespan (Morris *etal*., 2002; Ramirez *etal*., 2003) and carcinogen-treated mice carrying oncogenic CDK4 are highly susceptible to melanoma development (Sotillo *etal*., 2001). Our results demonstrate that ectopic expression of wild-type CDK4 overcame p16^INK4a^-induced arrest and senescence, and it is not surprising that overexpression of its homologue, CDK6, but not the downstream kinase CDK2, would abrogate p16^INK4a^ activity. Thus, the ability of p16^INK4a^ to bind and inhibit CDK4 and CDK6 is directly linked not only to cell cycle regulation but also to initiating the senescence programme.

The critical downstream target of the p16^INK4a^-CDK4/6 complex is Rb, which is strictly required for p16^INK4a^-mediated cell cycle arrest. Moreover, we now show that Rb is central to the p16^INK4a^-induced senescence programme, and that in the absence of Rb p16^INK4a^ does not promote SA-β-gal activity or chromatin condensation. Surprisingly, the status of Rb did not affect the ability of p16^INK4a^ to induce large increases in cellular size and extensive vacuolization, reminiscent of the morphological changes induced by various oncogenes (Denoyelle *etal*., 2006). The mechanism and impact of this p16^INK4a^ activity remains to be defined, although it is dependent on CDK4/6 inhibition and is presumably associated with the ability of p16^INK4a^ to elevate protein synthesis and ATP levels (Ausserlechner *etal*., 2005).

Although p16^INK4a^-induced cellular senescence provides an important brake to human cell transformation in culture its contribution to the tumour suppressor functions of p16^INK4a^ has been poorly defined. Our data confirm that senescence induction is tightly linked to the cell cycle inhibitory actions of p16^INK4a^, and importantly that both these functions are disabled by highly penetrant melanoma-associated variants. Furthermore, our data identify CDK4 and CDK6 as the central kinase targets of p16^INK4a^ in the regulation of senescence. Our results provide the first evidence that p16^INK4a^ can initiate an autonomous senescence programme that is disabled by inherited melanoma-associated mutations. This is consistent with the notion that the senescence programme limits the development of tumours and the inability to initiate and maintain senescence is an important contributor to melanoma development.

## Experimental procedures

### Cell culture and transfections

Human WMM1175 melanoma cells (ARF-null, p53-null, Rb^+/+^; Rizos *etal*., 1999) and HEK293T cells were grown in Dulbecco's modified Eagle's medium (DMEM, Gibco BRL, Carlsbad, CA, USA) supplemented with 10% foetal bovine serum and glutamine. Human epidermal melanocytes (HEM1259) were obtained from Cell Applications (San Diego, CA, USA) and grown in HAM's F10 media, supplemented with ITS premix (Becton Dickinson, Franklin Lakes, NJ, USA), TPA, IBMX, cholera toxin, 20% foetal bovine serum and glutamine (modified from Halaban *etal*., 1986). All cells were cultured in a 37 °C incubator with 5% CO_2_.

The WMM1175_p16^INK4a^ cell clones carrying the stably integrated *p16^INK4a^*(wild-type or mutant) gene under IPTG-inducible expression control has been described previously (Becker *etal*., 2001). The U20S_p16^INK4a^ cell clones were generated as previously described (Becker *etal*., 2001), except that a pooled population of transfected cells was analysed. p16^INK4a^ inducible cells were maintained in DMEM/10% foetal bovine serum supplemented with 250 µg mL^−1^ hygromycin and 500 µg mL^−1^ geneticin (Gibco). Stable cells were seeded 24 h prior to induction in the absence of antibiotics and were induced with 4 mm IPTG.

For *CDK2*, *CDK4*, *CDK6* and *Rb* transfections, cells (1 × 10^5^) were seeded on coverslips in six-well plates and transfected with 2 µg *CDK4-pEGFPN1*, *CDK6-EYFPN1*, *CDK2-HA*, *Rb*, *pEGFPN1* (Clontech, Mountain View, CA, USA) or pCMV-HA vector using Lipofectamine 2000 (Invitrogen, Carlsbad, CA, USA).

### Lentivirus transductions

Lentiviruses were produced in HEK293T cells using the *pSIH1-H1-copGFP* (Copepod green fluorescent protein) shRNA expression vector (Systems Biosciences, Mountain View, CA, USA) encased in viral capsid encoded by three packaging plasmids as described previously (Dull *etal*., 1998). Viruses were concentrated as described previously (Reiser, 2000). Viral titres were determined using 1 × 10^5^ U2OS cells/well in six-well plates, transduced with serial dilutions of the concentrated viral stocks in the presence of Polybrene (8 µg mL^−1^; Sigma, St. Louis, MO, USA). Cells were harvested 48 h post-transduction, analysed by flow cytometry for GFP expression and viral titre calculated.

For Rb silencing experiments, cells were transduced at an MOI of 10 with either a virus encoding Rb shRNA or a control shRNA, with no homology to any human gene. Cells were incubated for 72–96 h prior to analysis to allow expression of shRNA constructs and efficient silence of Rb.

### Constructs

*CDK6-EYFP* was constructed by subcloning the *CDK6* insert from *CDK6-PVL1292*(a gift from B. Sarcevic) into *pEYFPC1* (Clontech). The Rb-directed shRNA sequence corresponds to nucleotides 662–680 (GenBank accession number NM_000321.1). The control shRNA sequence 5′-TTAGAGGCGAGCAAGACTA-3′ showed no homology to any known human transcript.

### Western blotting

Total cellular proteins were extracted at 4 °C using RIPA lysis buffer containing protease inhibitors (Roche, Basel, Switzerland). Proteins (30–50 µg) were resolved on 12% sodium dodecyl sulfate–polyacrylamide gels and transferred to Immobilon-P membranes (Millipore, Bedford, MA, USA). Western blots were probed with antibodies against p16^INK4a^ (N20, Santa Cruz Biotechnology, Santa Cruz, CA, USA), p21^Waf1^ (C-19, Santa Cruz), Grp 78 (H129, Santa Cruz), total Rb (aa 332-344, Becton Dickinson), phosphorylated p-Rb^Ser807/811^ (#9308, Cell Signalling, Danvers, MA, USA), α-tubulin (236-10501, Invitrogen), β-actin (AC-74, Sigma-Aldrich), CDK4 (DCS-31, Sigma-Aldrich) and CDK6 (K6.90+K6.83, Neomarkers, Fremont, CA, USA).

### Proliferation assays

Cells were seeded at 1000 cells per well in a 96-well plate, with or without 4 mm IPTG. Number of viable cells was determined daily over 5-day induction period using the MTS assay (Promega, Madison, WI, USA) and analysed with the VICTOR^2^ 1420 Multilabel Counter (PerkinElmer, Waltham, MA, USA).

### Flow cytometry

For cell cycle analysis, cells were fixed in 70% ethanol at 4 °C for at least 1 h, washed in PBS and stained with propidium iodide (50 ng µL^−1^) containing ribonuclease A (50 ng µL^−1^). DNA content from at least 6000 cells was analysed using ModFIT software (Verity Software House, Topsham, ME, USA). The percentage of S-phase inhibition was calculated using the following formula: [(percentage of S-phase cells in uninduced cells) – (percentage of S-phase cells in induced cells)/(percentage of S-phase cells in uninduced cells)] × 100. Cell size and granularity was determined using flow cytometry on unfixed cells or cells fixed in 1% paraformaldehyde/PBS and analysed with CellQuest Pro (BD Biosciences)

### Indirect immunofluorescence

Cultured cells (3–4 × 10^4^) seeded on coverslips in 12-well plates were washed in PBS and fixed in 2% formaldehyde, 0.2% glutaraldehyde, 7.4 mm Na_2_HPO_4_, 1.47 mm KH_2_PO_4_, 137 mm NaCl, and 2.68 mm KCl. Cells were then rinsed three times with PBS and SA-β-gal activity was detected as previously described (Dimri *etal*., 1995). The same cells were immunostained for 50 min with primary antibodies followed by a 50-min exposure to Alexa Fluor 488- or Alexa Fluor 594-conjugated secondary IgG (Molecular Probes, Carlsbard, CA, USA). Nuclear DNA was stained with 1 µg mL^−1^ DAPI for 10–15 min.
